# The extended fronto-striatal model of obsessive compulsive disorder: convergence from event-related potentials, neuropsychology and neuroimaging

**DOI:** 10.3389/fnhum.2012.00259

**Published:** 2012-09-24

**Authors:** Margherita Melloni, Claudia Urbistondo, Lucas Sedeño, Carlos Gelormini, Rafael Kichic, Agustin Ibanez

**Affiliations:** ^1^Laboratory of Experimental Psychology and Neuroscience (LPEN), Institute of Cognitive Neurology (INECO) and Institute of Neuroscience, Favaloro UniversityBuenos Aires, Argentina; ^2^National Scientific and Technical Research Council (CONICET)Buenos Aires, Argentina; ^3^Laboratory of Cognitive and Social Neuroscience, Universidad Diego PortalesSantiago, Chile

**Keywords:** ACC, basal ganglia, conflict monitoring, ERN, ERPs, neuroimaging, OCD, OFC

## Abstract

In this work, we explored convergent evidence supporting the fronto-striatal model of obsessive-compulsive disorder (FSMOCD) and the contribution of event-related potential (ERP) studies to this model. First, we considered minor modifications to the FSMOCD model based on neuroimaging and neuropsychological data. We noted the brain areas most affected in this disorder -anterior cingulate cortex (ACC), basal ganglia (BG), and orbito-frontal cortex (OFC) and their related cognitive functions, such as monitoring and inhibition. Then, we assessed the ERPs that are directly related to the FSMOCD, including the error-related negativity (ERN), N200, and P600. Several OCD studies present enhanced ERN and N2 responses during conflict tasks as well as an enhanced P600 during working memory (WM) tasks. Evidence from ERP studies (especially regarding ERN and N200 amplitude enhancement), neuroimaging and neuropsychological findings suggests abnormal activity in the OFC, ACC, and BG in OCD patients. Moreover, additional findings from these analyses suggest dorsolateral prefrontal and parietal cortex involvement, which might be related to executive function (EF) deficits. Thus, these convergent results suggest the existence of a self-monitoring imbalance involving inhibitory deficits and executive dysfunctions. OCD patients present an impaired ability to monitor, control, and inhibit intrusive thoughts, urges, feelings, and behaviors. In the current model, this imbalance is triggered by an excitatory role of the BG (associated with cognitive or motor actions without volitional control) and inhibitory activity of the OFC as well as excessive monitoring of the ACC to block excitatory impulses. This imbalance would interact with the reduced activation of the parietal-DLPC network, leading to executive dysfunction. ERP research may provide further insight regarding the temporal dynamics of action monitoring and executive functioning in OCD.

## Introduction

Obsessive-compulsive disorder (OCD) affects approximately 50 million people worldwide (Sasson et al., 1997). According to the World Health Organization, OCD was estimated to be the 11th leading cause of non-fatal burden in the world in 1990 (Nestadt et al., 1998). It is characterized by the occurrence of obsessions (persistent and intrusive thoughts), compulsions (ritualistic behaviors aimed at reducing anxiety produced by obsession), or both (Franklin and Foa, 2011). The most common symptoms are symmetry/ordering, contamination/cleaning, checking behaviors, obsessions, and hoarding. OCD symptoms significantly reduce patients' quality of life (Fontenelle et al., 2010) and interfere with their routines and social life. OCD is a financial burden to both the individual and the health care system (Taylor, 2011). Although, several pharmacological and cognitive-behavioral treatments are currently available, a significant percentage of patients do not respond to them (Franklin and Foa, 2011). This failure underscores the limited understanding regarding the neurobiological mechanisms of OCD and highlights the need for further research.

Both psychodynamic (Freud, 1955) and cognitive-behavioral models (Salkovskis, 1999) have explained the disease using psychological hypotheses. More recently, neurocognitive studies have utilized different models to understand the brain networks involved. Despite some inconsistencies among neuropsychological and neuroimaging results (Friedlander and Desrocher, 2006; Maia et al., 2008; Clark et al., 2009), several findings have linked cognitive deficits to dysfunction in specific brain areas (Greisberg and McKay, 2003; Kuelz et al., 2004).

OCD functional imaging studies suggest abnormal metabolic activity in the orbitofrontal cortex (OFC), anterior cingulate cortex (ACC), and basal ganglia (BG) during rest or symptom provocation (Baxter et al., 1987; Breiter et al., 1996; Saxena et al., 1998; Kwon et al., 2003a). The OFC and ACC are intimately connected to the BG via cortico-basal ganglia-thalamo- cortical loops (Alexander et al., 1986; Mega and Cummings, 2001; Middleton and Strick, 2001). Based on this and other evidence, a model that implicates disturbed fronto-striatal brain systems in the pathogenesis of the disorder has been developed, referred to as the fronto-striatal model of obsessive-compulsive disorder (FSMOCD) (Baxter et al., 1987; Saxena et al., 1998; Mataix-Cols et al., 2004; Huey et al., 2008; Maia et al., 2008; Menzies et al., 2008; Chamberlain and Menzies, 2009; Clark et al., 2009; Simon et al., 2010; Freyer et al., 2011; Kuhn et al., 2011). Early versions of the FSMOCD were obtained from the cortico-striatal model which Alexander proposed in the 1980s. Nevertheless, the current literature refers to an extended and interconnected FSMOCD (Haber and Knutson, 2010; Milad and Rauch, 2012; Robbins et al., 2012). Specifically, the circuit connecting the OFC, the ventromedial caudate nucleus, additional substructures of the BG, and the thalamus, presents an imbalance of feedback loops (Kathmann et al., 2005). It has been suggested that each of these circuits play a relatively specific functional role based on the connections within each circuit to other frontal cortex areas (Menzies et al., 2008).

The circuits involved in the extended FSMOCD are modulated by dopaminergic, serotoninergic, noradrenergic, and cholinergic neurotransmitters (Dalley et al., 2008; Krebs et al., 2012). Given this frontal-basal connection and its modulation by neurotransmitters, fronto-striatal circuits appear to be essential for behavioral regulation (Menzies et al., 2008; Freyer et al., 2011). Indeed, the OFC is thought to be involved in motivational behavior, monitoring, and decision making. The ACC monitors actions and thoughts involving the detection of error and cognitive conflicts, and participates in reward processing [in OCD patients, ACC cingulotomy has been shown to reduce the symptomatology (Dougherty et al., 2002)]. Finally, the BG are involved in implicit learning, habit acquisition and action thresholds, and present an abnormal volume and activation in OCD (Graybiel and Rauch, 2000). Moreover, compulsivity has been proposed to reflect aberrant dysregulation of stimulus-response habit learning (Robbins et al., 2012).

Other models have proposed different areas to be altered during neuroimaging assessment of OCD, (Huey et al., 2008). The dorsolateral prefrontal cortex (DLPFC) is thought to be directly connected with the FSMOCD through the dorsolateral prefronto-striatal loop (Menzies et al., 2008). The DLPC and parietal cortex have been associated with executive function (EF) deficits [especially working memory (WM), Milad and Rauch, 2012]. Moreover, the DLPFC is reported to be involved in decision making, playing a coordinated role with the monitoring system (the ACC and the fronto-striatal circuit, Heekeren et al., 2008). In this work, we focus on the extended FSMOCD and related DLPC/parietal networks. Finally, we discuss the potential roles of other circuits (the ACC-amygdalo-cortical circuitry, the temporal lobes and other areas, such as the insula).

Numerous OCD reviews have focused on neuroanatomical models or on the fronto-striatal networks and their connections. Conversely, no single review has highlighted the importance of event-related potentials (ERPs) studies assessing action monitoring, inhibition, and other related executive functions, such as WM. The purpose of this review is to integrate the ERP findings (summarized in Table 1) with the extended FSMOCD. We first summarize the basis of the FSMOCD and its relationship with OCD cognitive deficits and neuroimaging results. Then, we analyze the possible connection between these findings and ERP results. Finally, we examine the convergence of these discoveries and methodologies, and discuss future directions of research.

**Table 1:** **Representative studies of ERN, N2 and P600**.

**Author**	**Participants**	**Comorbidity**	**Medication**	**Paradigm**	**Main results**	**Multivariate comparison and/or additional measures?**
**ERN STUDIES**						
Gehring et al., 1993	6 HS	NR	NR	FT	ERN activity is enhanced when subjects strive for accurate performance but is diminished when subjects aim for response speed instead of accuracy	NO
Gehring et al., 2000	9 OCD, 9 controls	7 = MD, PDA, AN, SP, AB. 2 = D, SO. 1 = PD, GA	Fluoxetine (2), Clomipramine (1), Sertraline (3)	Modified ST	Enhanced ERN in OCD patients. Correlates with symptom severity	NO
Johannes et al., 2001	10 OCD, 10 controls	No history of MD or SP	Not pharmacologically treated	A reaction time experiment	Enhanced ERN in OCD patients	P3b
Hajcak and Simons, 2002	18 HOCD, 17 LOCD	NR	NR	Modified ST	Enhanced ERN in OCD patients but no differences in performance between groups	NO
Hajcak et al., 2005	22 HS (exp.1), 18 HS (exp. 2)	NR	NR	FT with low/high value errors (exp. 1) –and evaluation/control conditions (exp. 2)	ERN was significantly larger on high-value trials in both experiments	NO
Hajcak et al., 2008	Pre: 18 POCD, 18 controls//Post: 10 POCD, 13 controls	NR	Clomipramine (2), Sertraline (3), Escitalopram (4), Fluoxetine (1), Bupropion (2), Fluvoxamine (1)	Pre and post evaluation after cognitive behavior therapy. Modified simon task	ERN was larger in pediatric OCD patients before and after treatment. There was no relationship between ERN and symptom severity	NO
Endrass et al., 2008	20 OCD, 20 controls	AD (8), GA (5), PD(4)	Clomipramine (4), Paroxetine (2), Fluoxetine (1), Fluoxetine plus trimipramine (1), Venlafaxine (2)	FT (modified version)	OCD patients showed enhanced ERN amplitude on both error and correct trials. CRN amplitude correlates with symptom severity	CRN, Pe, behavioral correlations of performance monitoring
Endrass et al., 2010	22 OCD, 22 controls	MD (2), GA (2), PD (3)	Clomipramine (2), Sertaline (1), Fluoxetine (2), Mirtazapine (1), Fluvoxamine (1)	FT (modified version with standard and punishment conditions)	In the standard condition OCD patients had significantly larger ERN and CRN amplitudes than controls. No differences were found in the punishment condition. Controls showed an amplitude enhancement between standard and punishment conditions, while OCD patients did not	CRN, Pe
Grundler et al., 2009 (Study I)	10 HOCD, 30 LOCD	NR	NR	PRT	Higher OCD symptoms predicted smaller ERNs	NO
Grundler et al., 2009 (Study II)	14 HOCD, 16 LOCD (PRT)//18 HOCD, 18 LOCD (FT)	NR	NR	PRT, FT	High OCD group presented smaller ERN in a probabilistic task and larger ERN in a flanker task	NO
Bernstein et al., 1995	30 HS	NR	NR	A four-choice reaction time task	ERN was no longer than expected	NO
Falkenstein et al., 2000	24 HS	NR	NR	GN, FT	ERN had similar amplitude in tasks with a strong response conflict and tasks without any such conflict. ERN activity was found on correct trials	Pe
Van Veen and Carter, 2002b	12 HS	NR	NR	FT	Reported significant differences between correct and error waveforms indicate that the ERN is significantly more negative than the waveform following correct responses	N2, Pe
Holroyd and Coles, 2002: Experiment 1	15 HS	NR	NR	PRT	ERN tended to be larger when the feedback stimulus disconfirmed, rather than confirmed, a prediction induced by a previous feedback stimulus	Psychophysiological experimentation and computational modeling
Holroyd and Coles, 2002: Experiment 2	15 HS	NR	NR	FT feedback informs the participants their accuracy and average speed	ERN amplitude was larger on frequent incompatible error trials than on infrequent compatible and infrequent incompatible error trials	Psychophysiological experimentation and computational modeling
Nieuwenhuis et al., 2005	16 OCD, 16 controls	NR	Paroxetine (5), Clomipramine (1), Citalopram (1), Fluvoxamine (1), Venlafaxine (1), Clonazepam (1)	PRT	The amplitude of the ERN associated with error and negative feedback was the same for OCD patients and controls	NO
Santesso et al., 2006	37 health children	NR	NR	FT	Parent-reported obsessive-compulsive behaviors were associated with larger ERN	CBCL, Pe
(De Bruijn et al., 2004)	12 HS	No neuropsychiatric conditions	Medication free	FT	Amphetamine led to a strong enlargement of ERN amplitudes without affecting reaction times. Lorazepam led to reduced ERN amplitudes	D-amphetamine, lorazepam, mirtazapine or placebo was administered in a double-blind, four-way crossover design.
Riba et al., 2005a	15 HS	Medical history, laboratory tests, electrocardiogram and urinalysis were normal	Medication free	FT	Yohimbine (adrenoceptor antagonist) led to both an increase in ERN amplitude and a significant reduction in action errors	20 mg of yohimbine and a placebo were administered a double-blind randomized design (DBRD)
Riba et al., 2005b	12 HS	Medical history, laboratory tests, electrocardiogram and urinalysis were normal	Medication free	FT	Alprazolam significantly reduced the amplitude of ERN and the number of correct responses and increased reaction time	N2, LRPs // Oral doses of 0.25 and 1.0 mg of alprazolam or placebo were administered in a DBRD
Anokhin et al., 2008	Twins: 99 MZ and 175 DZ	Medical history was normal	Medication free	FT	Substantial heritability of ERN, CRN and Pe (40–60%), ERP showed significant genetic correlations among them	CRN, Pe
Riesel et al., 2011	30 OCD, 30 UFO, 30 HS	OCD: MD (4), SO (3), PD (1), GA (1), SP (2), BN (1), PD (3)	OCD: Selective serotonin reuptake inhibitors (7), Tricyclic antidepressants (3)	FT	Both unaffected first-degree relatives and OCD patients showed increased ERN. ERN did not correlate with symptom severity	CRN
Ridderinkhof et al., 2002	14 SD	No history of neurological or psychiatric condition	Medication free	FT	The consumption of alcohol in moderate doses reduced participants' task error detection and ERN/N200 amplitudes	A double-blind, placebo-controlled, randomized cross-over design
Holroyd et al., 1998	15 HS	NR	NR	FT	ERN is generated within the ACC	NO
Miltner et al., 2003	6 HS	NR	NR	GN, MG	Magnetic equivalent of the ERN and dipole source analysis evidenced ACC generators	NO
Stemmer et al., 2004	5 LACC, 11 controls	NR	NR	FT	Implication of the rostral ACC in ERN generation and also the results show that although subjects can be aware of errors, no ERN is produced	NO
N200 AND P600 STUDIES						
Ciesielski et al., 2011	9 OCD, 9 controls	NR	NR	ST-WCIT (a high conflict variant)	Enhanced N200 amplitude and normal accuracy in OCD patients	NO
Kopp et al., 1996b	18 HS	NR	NR	FT	The incongruent condition elicited a N200 component synchronized with an erroneous response. N200 amplitude covaried with the magnitude of the erroneous response	NO
Liotti et al., 2000	8 HS	No history of neurological or psychiatric illness	NR	ST	ST first activates anterior cingulated cortex (350–500 ms post-stimulus) followed by activation of the left temporal-parietal cortex, possibly due to the need for additional processing of word meaning	NO
Wang et al., 2000	15 HS	No history of neurological or psychiatric illness	NR	arithmetic problem and answer digit matched task	N200 elicited by incongruence among stimuli, while N270 evoked by a physical feature discrimination task and conflict or mental mismatching	N270
Yeung et al., 2004	16 HS	NR	NR	FT	ERN and N2 shared a very similar scalp topography and neural source	ERN
Kopp et al., 1996a	18 HS	NR	NR	Hybrid choice-reaction GN involving selective response priming	In no-go trials the N2 amplitude was influenced by selective response priming. The N2 was elicited in both go and no-go trials	LRP, P3
Heil et al., 2000	18 HS	NR	NR	GN, FT	Target and flankers were assigned to different hands. The flankers primed by one hand were accompanied by a fronto-central amplitude modulation of the N200	LRPs
Eimer, 1993	6 HS	NR	NR	A modified GN	No-go stimuli elicited larger N2 components than go stimuli. The N2 enhancement showed a frontal maximum	P3s
Beech et al., 1983	8 OCD, 8 controls	NR	Antidepressant medication was stopped 48 h before testing (3)	A task of varying complexity involving shape discrimination	Reduced amplitudes and decreased latencies of late EP components (N220 and P350) in OCD patients	P3
Towey et al., 1993	17 OCD, 16 HS	Absence of major medical problems	Drug free for at least 2 weeks before testing	Auditory “oddball” stimuli	Lager N200 and P3 in OCD patients. Task difficulty increased N200 latencies for controls, but not for OCD patients	P300.
Papageorgiou and Rabavilas, 2003	20 OCD, 20 HS	Exclusion criteria: MD, GA	Drug free for at least 3 weeks for the time of evaluation	WST (Computerized version)	Enhanced amplitudes of P600 at the right temporoparietal area and prolonged latencies at the right parietal region in OCD patients. Memory performance was also significantly impaired	NO

**Participants** HS, Healthy subjects; OCD, Obsessive-compulsive disorder patients; HOCD, subjects with high OC symptoms (but no OCD patients); LOCD, subjects with low OC symptoms (but no OCD patients); POCD, pediatric OCD; MZ, monozygotic; DZ, dizygotic; UFO, unaffected first degree relatives of OCD; SD, social drinkers (2–3 units per day on average); LACC, lesions in the ACC; FG, patients with lesions outside the frontal cortex; BG, lesions to the basal ganglia; OCT, orthopedic controls.

**Comorbidity** MD, major depression; PDA, panic disorder with agoraphobia; NA, anorexia nervosa; BN, bulimia nervosa; SP, specific phobia; AB, alcohol abuse; D, dysthymia; SO, social phobia; PD, panic disorder without agoraphobia; GA, generalized anxiety disorder; AD, affective disorder; PD, personality disorder.

**Others** NR, not reported in the paper; FT, Flanker Task; ST, Stroop Task; PRT, probabilistic reinforcement learning task; GN, Go/No go Task; WST, Wechsler digit span test; MG, magneto encephalography; CRN, correct related negativity; Pe, error positivity; LRPs, lateralized readiness potentials; CBCL, parents report form designed to assess children behaviors.

## Neuropsychology of OCD

The OCD neuropsychological profile can be characterized by deficits in two domains: planning (Greisberg and McKay, 2003) and the inhibition of motor/cognitive intrusive or inappropriate behaviors (Chamberlain et al., 2005). Both of these alterations and the reported impairments in memory, attention and visuospatial abilities suggest problems in executive functions (Martinot et al., 1990; Savage et al., 1999; Deckersbach et al., 2000; Savage and Rauch, 2000; Kim et al., 2002; Van Der Wee et al., 2003; Kuelz et al., 2004).

### Response inhibition and set shifting

OCD cognitive deficits include failure to inhibit or shift attention from intrusive thoughts or motor activities toward more pleasant ones (Chamberlain et al., 2005). In the Go/No Go task (see reviews Chamberlain et al., 2005; Chamberlain and Menzies, 2009) OCD patients make more commission errors (Bannon et al., 2002; Aycicegi et al., 2003; Milad and Rauch, 2012), showing that they tend to exhibit inappropriate motor responses to non-target stimuli.

To examine set-shifting abilities in OCD, a number of investigations have employed the Wisconsin Card Sorting Test (WCST; Berg, 1948), the Object Alternation Test (OAT; Freedman, 1990) and/or the Delayed Alternation Test (DAT; Freedman and Oscar-Berman, 1986). Although some studies have reported impaired performance of OCD patients in the WCST (Boone et al., 1991; Hymas et al., 1991; Lucey et al., 1997a; Okasha et al., 2000), most investigations suggest that OCD subjects' results are similar to those of healthy controls (Zielinski et al., 1991; Abbruzzese et al., 1995a,b, 1997; Gross-Isseroff et al., 1996; Deckersbach et al., 2000; Moritz et al., 2001a,b, 2002). However, investigations using the OAT and DAT have found marked deficits in these patients compared to controls (Abbruzzese et al., 1995a, 1997; Gross-Isseroff et al., 1996; Cavedini et al., 1998; Moritz et al., 2001b; Spitznagel and Suhr, 2002). The last two tests have been suggested to be sensitive to OFC damage (Freedman et al., 1998), and increased activity of the OFC during the performance of these tasks has been reported (Zald et al., 2002). In contrast, the WCST does not engage one specific brain area but involves a distributed neural network (Posner and Petersen, 1990; Fernandez-Duque and Posner, 2001; Barcelo, 2003).

In summary, OCD patients present deficits in the inhibition processes and show impaired attention shifting during some neuropsychological tasks. Based on these deficits, Chamberlain et al. (2005) proposed that people with OCD may exhibit impairments in (1) cognitive inhibition, and (2) behavioral inhibition. This is a conceptual distinction that can be useful for understanding patient behavior.

### Planning capacity

Impaired planning capacity has been reported in OCD patients (Cavedini et al., 2001; Nielen and Den Boer, 2003; Van Den Heuvel et al., 2005) and subclinical obsessive-compulsive participants (Mataix-Cols et al., 1999) (but see Veale et al., 1996; Purcell et al., 1998 for contradictory results regarding accuracy).

Evidence from the investigation of other cognitive domains supports the existence of this OCD planning deficit. Memory dysfunction is associated with information organization at encoding and/or retrieval (Kuelz et al., 2004; Chamberlain et al., 2005). Impaired recall performance on non-verbal memory tests is due to the failure to employ appropriate organizational strategies (Martinot et al., 1990; Savage et al., 1999; Deckersbach et al., 2000; Savage and Rauch, 2000; Kim et al., 2002; Kuelz et al., 2004). Verbal memory is not impaired in OCD patients (Christensen et al., 1992; Martin et al., 1995; Mataix-Cols et al., 1999), except on tests that require semantically clustering the stimuli and responses (Savage and Rauch, 2000; Cabrera et al., 2001). OCD patients perform worse than controls in spatial WM tasks that are strategy-dependent (Van Der Wee et al., 2003). Finally, the visuospatial difficulties observed in OCD patients might also be related to EF deficits (Head et al., 1989; Christensen et al., 1992; Tallis, 1997a). These results suggest that both memory and visuospatial impairments are, in fact, indexing deficits in other areas, such as strategy processing, set-shifting and/or processing speed.

### Summary of neuropsychological findings

The high variability observed in the neuropsychological profile can be partially explained by both the cognitive heterogeneity involved in OCD (Chamberlain et al., 2005) and possible confounding factors [psychotropic medication, symptom severity, education, and co-morbidity (Kuelz et al., 2004)]. Nevertheless, the overall neuropsychological evidence suggests that, secondary to impairments in cognitive strategies; both executive and more general action-monitoring deficits are present.

## Neuroimaging findings in OCD: the role of the OFC, ACC, and BG

**Figure 1:**
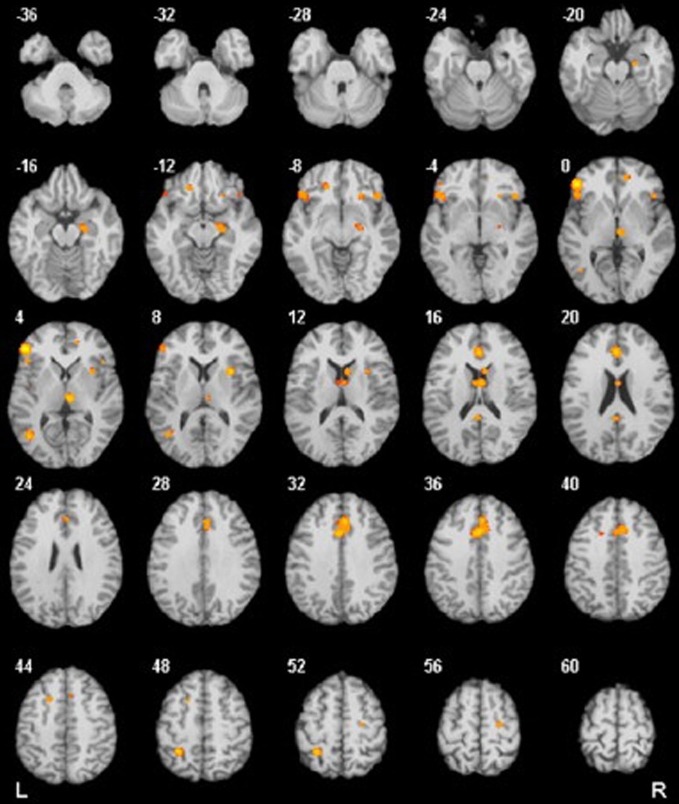
**Results from a quantitative voxel-level meta-analysis of fMRI studies reporting case-control differences associated with OCD across a range of paradigms**. The panels depict areas where activation was greater in OCD patients than in healthy controls (*p* < 0.05). The R and L markers denote the side of the brain, and the numbers denote the *z* dimension of each slice in MNI space. The activation of these areas is consistent with the areas involved in the FSMOCD described in the present review. Reproduced with authorization from Neuroscience and Biobehavioral Reviews (Menzies et al., 2008).

Several studies reported increased metabolism and hyperactivity in several areas in OCD patients, including the BG (Swedo et al., 1989; Baxter et al., 1992; Molina et al., 1995; Perani et al., 1995), OFC (Baxter et al., 1988; Molina et al., 1995; Alptekin et al., 2001), and ACC (Swedo et al., 1989; Molina et al., 1995; Perani et al., 1995) cortices (see Figure 1). Furthermore, there is evidence of decreased activation in the DLPFC and parietal cortex during symptom provocation (Maltby et al., 2005; Van Den Heuvel et al., 2005; Viard et al., 2005; Remijnse et al., 2006). These brain areas have been associated with OCD neurocognitive deficits and symptoms as well as with functional connectivity of the fronto-striatal system. Here, we summarize the main findings on this topic.

### OFC and BG

BG and OFC hyperactivation constitutes an interconnected neural circuit that may be involved in obsessions and compulsive actions (Graybiel and Rauch, 2000). Zald and Kim (1996) described the OFC as an interface area between sensory association cortices, limbic structures, and subcortical regions (BG) involved in the control and monitoring of autonomic and motor effector pathways. Several authors (Tremblay and Schultz, 1999; Elliott et al., 2000; Tremblay and Schultz, 2000; Hikosaka and Watanabe, 2004) have suggested that OFC ascribes and monitors changes in reward value. These OFC functions (monitoring changes and withholding previously learned actions) suggest that this region may play an inhibitory role in certain behaviors.

Another structure related to behavioral regulation and flexibility is the BG, which interacts closely with the OFC. Graybiel and Rauch (2000) found that this area influences both motor and cognitive planning in OCD. According to Mercadante et al. (2004), in OCD patients, the BG does not filter cortical impulses (motor or cognitive activity) properly, which causes changes in thalamic activity. Some reports (Saxena et al., 1998, 2001) have proposed that there is an imbalance between the excitatory role of the BG and inhibitory overactivity of the OFC. This conflict might lead to obsessive thoughts and compulsive behaviors. For a more detailed view on the roles of the OFC and BG in OCD, see Milad and Rauch (2012); Robbins et al. (2012).

### ACC

In OCD patients, the ACC has been found to be hyperactive at rest, during symptom provocation and during performance of high-conflict cognitive tasks (Swedo et al., 1989; Machlin et al., 1991; Perani et al., 1995). This hyperactivity has been suggested to be associated with an excessive evaluation of performance in OCD, leading to inappropriate doubting, the need for correction and consequently, repetitive actions (Ursu et al., 2003; Fitzgerald et al., 2011).

ACC activation has often been associated with action selection and performance monitoring (Aouizerate et al., 2004). Some authors (Van Veen and Carter, 2002a; Ursu et al., 2003) suggest that a more specific function of this region is activation in response to conflicts occurring between incompatible streams of information processing (conflict theory). Following conflict detection, regions of the lateral prefrontal cortex and other areas associated with attention control are engaged to resolve the conflict (Van Veen and Carter, 2002a). As mentioned above, studies have reported ACC hyperactivation in OCD patients, suggesting that these regions are unusually sensitive to information conflict.

In OCD patients, ACC hyperactivity might indicate an overactive conflict response monitoring system. According to this hypothesis, patients could frequently over-evaluate possible responses against conflictive situations. This over-evaluation is in accord with their unimpaired performance in most cognitive tasks, although they take longer than controls to finish or solve the task. This hypothesis offers a possible explanation for OCD patients' constant doubting and repetition, despite accurate performance (Ursu et al., 2003).

### Dorsolateral prefrontal areas and parietal cortex

Evidence of decreased activation of frontal areas other than the OFC-BG and ACC has been reported during symptom provocation in OCD patients (Maltby et al., 2005; Van Den Heuvel et al., 2005; Viard et al., 2005; Remijnse et al., 2006). In particular, the DLPFC is implicated in executive planning (Menzies et al., 2008). Van Den Heuvel et al. (2005) observed DLPFC dysfunction together with impaired performance of OCD patients in the Tower of London Task. These authors suggested that the decreased responsiveness of the DLPFC is related to cognitive impairments in spatial attention and WM processes in OCD (Van Der Wee et al., 2003). Structural studies have also shown a decreased DLPFC volume in these patients (Martinot et al., 1990; Lucey et al., 1997b).

The parietal cortex is important for executive tasks involving functions such as attention, spatial perception, and WM (Cabeza and Nyberg, 2000; Culham and Kanwisher, 2001). Because executive functions and WM are domains that are relatively affected in OCD, it is possible that DLPC/parietal lobe network dysfunction contributes to OCD cognitive deficits. Posner and Petersen (1990) suggested that this region operates as part of a posterior attention system involved in disengaging spatial attention. Furthermore, it has been reported that activity in this lobe is related to sustaining attention and attention set-shifting (Nagahama et al., 1996; Le et al., 1998; Hampshire and Owen, 2006). The parietal lobe has also been implicated in planning (Williams-Gray et al., 2007) and response inhibition (Rubia et al., 2001; Lepsien and Pollmann, 2002; Horn et al., 2003), which are reported to be impaired in OCD.

Several studies (Cavada and Goldman-Rakic, 1989; Romanski et al., 1997; Roberts et al., 2007) have demonstrated connections between parietal regions and the DLPFC and determined that both regions contribute to the dorsolateral prefrontal-striatal circuit. Thus, OCD patients present dysfunction in both the orbitofrontal-striatal and dorsolateral prefrontal-striatal circuits.

### Summary of neuroimaging studies

The studies described above suggest (1) hyperactivation of the OFC, ACC, and BG involved in the control, monitoring and inhibition of behaviors and thoughts; and (2) decreased activity of the DLPC/parietal network involved in executive functions (attention and WM). Thus, an extended FSMOCD involving OFC/ACC/BG hyperactivation as well as DLPC/parietal deactivation suggests an intertwining of monitoring/inhibitory control with executive functions. These abnormalities in different areas might underlie the variety of OCD symptoms.

## ERP studies related to the FSMOCD

ERPs constitute a millisecond-by-millisecond record of neural information processing that can be associated with particular operations, such as sensory encoding, motor control or higher cognitive processes (Ibanez et al., 2012b). Thanks to its temporal resolution, ERP studies can accurately measure brain dynamics that occur during cognition (Picton et al., 2000). This represents an advantage over fMRI, which lacks such temporal resolution. However, the spatial resolution of ERP measurements is limited. Nevertheless, multichannel recordings allow investigators to estimate the intracerebral locations of cognitive processes (Picton et al., 2000). Therefore, ERP assessment has become an important tool with the potential to be used for studying sensory, motor, cognitive, and social processes (Ibanez et al., 2006, 2008, 2010a,b, 2011a,b, e, 2012b; Cornejo et al., 2009; Hurtado et al., 2009; Aravena et al., 2010; San Martin et al., 2010; Dufey et al., 2011; Ibáñez et al., 2012) and to provide neuropsychiatric biomarkers (Guerra et al., 2009; Ibanez et al., 2011c, d, 2012a,c; Ibáñez et al., 2011, 2012). In the following section, we present the ERP components that can be most directly related to the extended FSMOCD (see Table 1).

### Error-related negativity (ERN)

**Figure 2:**
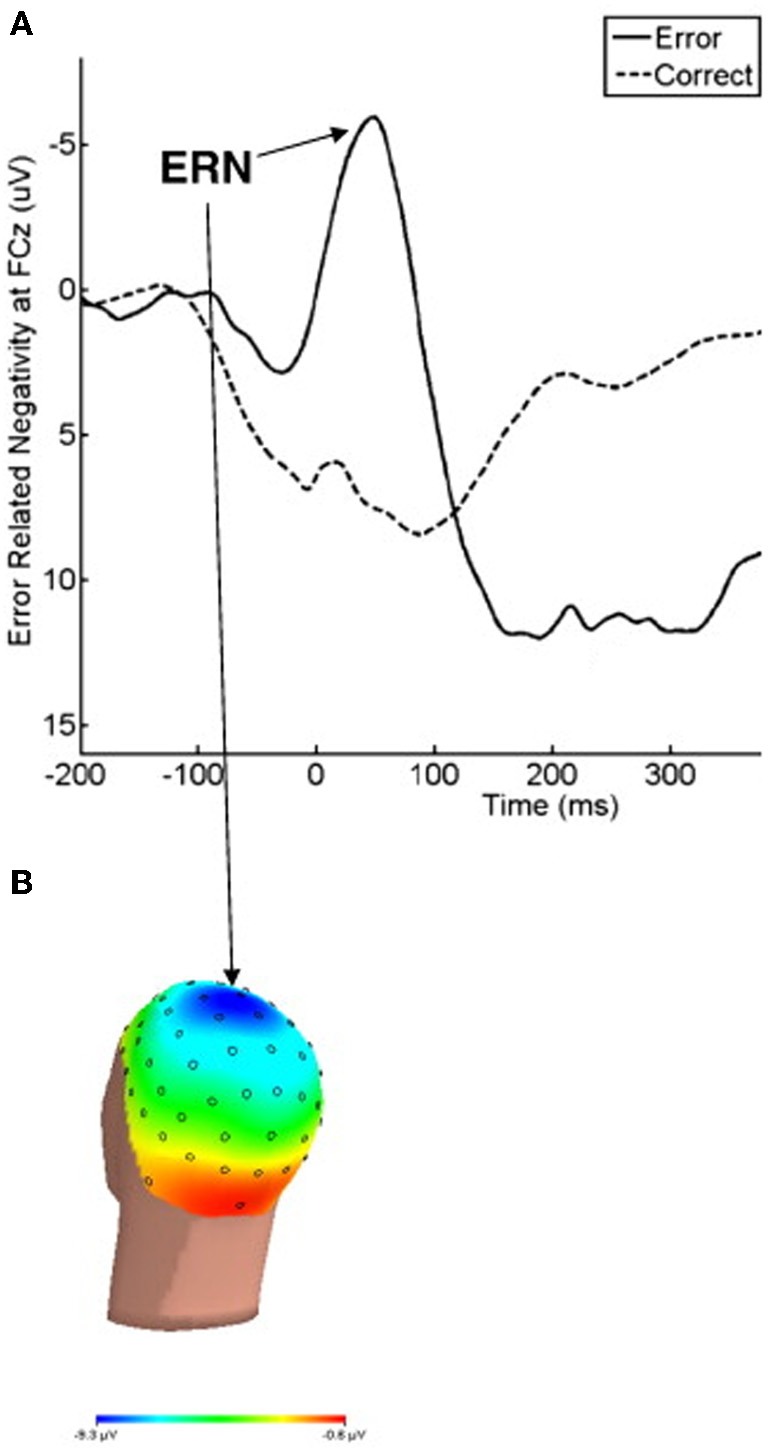
**(A)** The response-locked ERPs for error and correct trials at FCz, where ERN was maximal. **(B)** The response onset occurred at 0 ms, and negative values are plotted upward. Scalp topography of error-related brain activity from 0 to 100 ms post-response. Reproduced with authorization from Clinical Psychology Review (Olvet and Hajcak, 2008).

Errors during rapid response tasks are typically followed by a large ERP component known as error-related negativity (ERN) (Van Veen and Carter, 2006). This component is a negative deflection that occurs between 50 and 100 ms after having committed an error (see Figure 2). Several lines of evidence from different types of studies (source localization: Dehaene et al., 1994; Holroyd et al., 1998; Van Veen and Carter, 2002b; magnetoencephalography: Miltner et al., 2003; intracerebral recordings: Brazdil et al., 2005; ACC lesions with diminished ERN: Stemmer et al., 2004) support the idea that ERN is mainly generated in the ACC. In the above section, we discussed neuroimaging studies that reported hyperactivity in this area in OCD patients. Most of these fMRI investigations can be complemented with ERP measurement methods to improve the temporal resolution of their results.

#### ERN: the debate about its functional significance

Although subjects with OCD show increased ERN amplitude compared to control subjects (Gehring et al., 2000; Johannes et al., 2001; Hajcak and Simons, 2002; Santesso et al., 2006; Endrass et al., 2008; Hajcak et al., 2008; Grundler et al., 2009; see also the ERN meta-analysis in Mathews et al., 2012), there is an extensive discussion about the functional significance of enhanced ERN amplitudes (Gehring et al., 2000; Johannes et al., 2001; Hajcak and Simons, 2002; Santesso et al., 2006; Endrass et al., 2008; Hajcak et al., 2008; Grundler et al., 2009). One of these theories suggests that ERN reflects a process that compares a representation of an intended response to a representation of the actual response (Falkenstein et al., 1991; Scheffers et al., 1996). Investigations that tested this hypothesis found that subjects exhibit larger ERNs when the error response and the correct response are more dissimilar (Bernstein et al., 1995; Falkenstein et al., 1995; Scheffers et al., 1996). However, other authors who support a different theory of ERN (conflict theory) argue that they have found many instances of ACC activation during correct trials. These results are in disagreement with the first hypothesis because ERN amplitude increased should be seen only for incorrect responses.

According to *conflict theory* (Van Veen and Carter, 2006), the ACC monitors the presence of conflict between two incompatible information-processing streams. From this point of view, ERN reflects the conflict between the fast erroneous responses and the slower correct ones (Botvinick et al., 1999; Van Veen and Carter, 2002b).

In error trials, conflict immediately follows an erroneous response. During interface tasks, conflict precedes the actual response in correct high-conflict trials (Van Veen and Carter, 2002b). These trials are frequently characterized by a small but rapid activation of the incorrect response and a slower activation of the correct one (Kopp et al., 1996b). Thus, during correct trials, the conflict between the initial incorrect activation and the overriding correct response takes place before the correct response (Van Veen and Carter, 2006). In conclusion, the “conflict theory” suggests that conflict occurs prior to the response in correct, high-conflict trials but follows the response in error trials (Van Veen and Carter, 2002b).

A third theory regarding the functional significance of ERN was presented by Holroyd and Coles and is known as “reinforcement learning theory” (Holroyd and Coles, 2002). The authors propose that behavior is monitored by a basal ganglia-based “adaptive critic” that determines whether events are better or worse than expected and signals this with a phasic increase or a decrease in ACC dopaminergic activity (Van Veen and Carter, 2006). Therefore, the function of this brain area is selection between different cognitive processes competing for access to the motor system. To fine-tune the ACC for more appropriate selection in future trials, the inhibitory influence of the dopaminergic innervations in the ACC is briefly disrupted, and it is assumed that this generates ERN. These authors based their findings on an ERP component that somewhat resembles ERN and appears to be elicited by error feedback stimuli and stimuli indicating loss or punishment (e.g., San Martin et al., 2010). Reward-based learning modifies both components; in a task in which participants had to learn the correct stimulus-response mapping by processing feedback stimuli, both components were observed to behave more or less as predicted (Holroyd and Coles, 2002). However, there is no sufficient evidence to support the assumption that this component has the same functional significance as the response-linked ERN.

Despite the theoretical discrepancies, all of these theories relate ERN to different aspects of monitoring behavior. There are convergent results showing an ERN increased amplitude in OCD patients. The evidence that supports ERN as an index of monitoring behavior, together with the enhancement of this component in OCD, might suggest that these patients exhibit subtly altered cognitive monitoring processes. These putative alterations have received different explanations. In the first theory, which suggests that ERN reflects the comparison between representations of an intended response and representations of the actual response, OCD subjects might present a hyperactive error monitoring system that induces a comparator dysfunction (Gehring et al., 2000). This hyperactivity could explain the tendency of patients to feel that something is wrong when the situation seems satisfactory to an outside observer. This dysfunction might cause seemingly correct repetitive actions to elicit error signals. However, “conflict theory” suggests that ERN amplitude increase, in both incorrect and high-conflict correct trials, indicates an overactive conflict monitoring system in OCD patients (Botvinick et al., 1999; Van Veen and Carter, 2002b). According to this theory, patients could be frequently over-evaluating possible conflict responses during motor and/or cognitive activities. Their overactive conflict monitoring system causes them to adopt a very cautious approach to test performance to avoid mistakes. This approach offers a possible explanation for the patients' constant doubting and need for repetitive action, despite correct performance (Ursu et al., 2003). The last theory (Holroyd and Coles, 2002) posits that ERN is the result of ACC disinhibition provoked by a dopamine decrease due to an event that did not turn out as expected. Once the erroneous action is performed, it is assumed that the subject must avoid making the same mistake again. Therefore, in OCD, ERN could be higher because subjects not only perceive the error but also present compulsory behaviors and excessive monitoring to avoid mistakes. Moreover, psychopharmacological investigations have demonstrated possible effects of medication in OCD, given that psychotropic drugs alter ERN (Ridderinkhof et al., 2002; De Bruijn et al., 2004; Zirnheld et al., 2004; Riba et al., 2005a,b).

#### A different point of view: error significance

Endrass et al. (2010) examined whether overactive performance monitoring in OCD patients is adjusted based on error significance and hypothesized that these patients are less able to monitor and correct their performance. In this study, the author found that healthy subjects' performance monitoring is strengthened under conditions with a higher error relevance or salience. This finding is supported by research detecting ERN enhancement when accuracy is emphasized over speed (Gehring et al., 1993; Falkenstein et al., 2000), when errors are associated with a high monetary risk or when errors are committed during social evaluation (Hajcak et al., 2005). To evaluate whether OCD patients show the same sensitivity to error relevance, Endrass et al. (2010) used a flanker task to compare patients and controls under two different conditions, one of which was neutral, while the other involved punishment feedback (in which error relevance is higher). The author reported three important results: (1) in the neutral condition, patients presented greater ERN amplitude than controls in both correct and incorrect trials; (2) there were no significant differences between groups under the punishment condition; and (3) controls showed amplitude enhancement between neutral and punishment conditions, whereas OCD patients did not show variations.

These results replicated earlier findings and support the interpretation that the ERN is sensitive to the motivational significance of errors (Hajcak et al., 2005). Additionally, the evidence that OCD patients demonstrated only overactive performance monitoring (vs. controls) in situations with a lower error significance and not in the punishment condition suggests that they are not as sensitive as normal subjects to error significance.

Furthermore, when OCD patients had reached maximum monitoring activity in the neutral condition due to a ceiling effect, they were unable to further increase monitoring activity (Endrass et al., 2010). This observation should explain some inconsistent results from probabilistic learning tasks (Nieuwenhuis et al., 2005; Grundler et al., 2009). During this type of task, these studies did not find significant differences in the ERN amplitude between OCD patients and healthy controls. However, Grundler et al. (2009) did find larger ERNs in flanker tasks for participants with increased OCD symptoms. According to this result, Endrass et al. (2010) suggested that compared to flanker tasks, the error significance might be higher during probabilistic learning tasks because subjects have to carefully attend to feedback to learn and predict correct responses. Therefore, the absence of group differences between OCD patients and healthy controls in these highly demanding tasks might be caused by the enhancement of monitoring activity in controls to a similar level as that in OCD patients.

#### ERN as a possible endophenotype for OCD

Endophenotypes are unobservable characteristics that mediate relationships between genes and behavioral phenotypes (Gottesman and Gould, 2003). The aim in searching for endophenotypes is to identify neural and information processing abnormalities that may place individuals at risk for developing psychopathology (Olvet and Hajcak, 2008).

**Figure 3:**
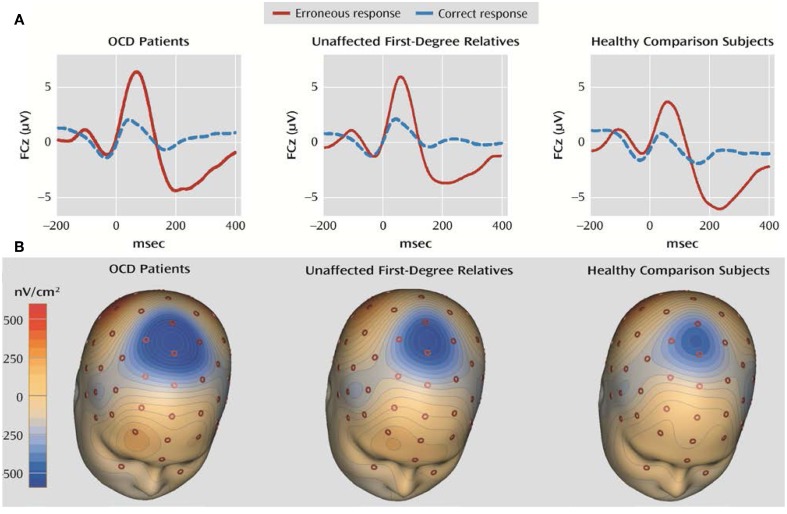
**Grand averages of EEG recordings and the error-related negativity topography in OCD patients, unaffected first-degree relatives of OCD patients, and healthy subjects for comparison**. **(A)** Response-locked grand average waveforms recorded at electrode FCz for correct and incorrect responses are shown. Responses occurred at 0 ms. **(B)** The ERN topography of all three groups is presented. The ERN is characterized by a fronto-central distribution with a maximum at electrode FCz. The current source density (latency 66 ms) was computed by Laplace transformations on grand average waveforms in the three groups. Modified from American Journal of Psychiatry (Riesel et al., 2011).

ERN is considered to be a potential suitable OCD endophenotype (Riesel et al., 2011) because it fulfills the criteria described in the scientific literature (Gottesman and Gould, 2003): (1) it is associated with OCD in that enhanced ERN amplitudes have been repeatedly found in patients with this disorder (Gehring et al., 2000; Johannes et al., 2001; Hajcak and Simons, 2002; Santesso et al., 2006; Endrass et al., 2008; Hajcak et al., 2008; Grundler et al., 2009); (2) it is heritable, as a twin study showed substantial ERN heritability of between 45% and 60% (Anokhin et al., 2008); (3) ERN amplitude enhancement appears to be independent of the symptom state in pediatric OCD patients (Hajcak et al., 2008); and (4) asymptomatic relatives of OCD patients who were not taking psychotropic medication showed enhanced ERN amplitudes similar to those of patients, as reported by an ERP investigation (see Figure 3) (Riesel et al., 2011).

The results of these studies on ERN and OCD suggest that enhanced ERN might be a candidate endophenotype for this disorder. Nevertheless, these findings are not sufficient to discern whether an enhanced ERN amplitude is a mediator between genes and an OCD phenotype or only a risk indicator associated with some of the same genes as this disorder (Kendler and Neale, 2010). In addition, endophenotypes might also be influenced by environmental risk factors that affect both the endophenotype and the clinical phenotype (Kendler and Neale, 2010). Further studies are needed to determine whether this component is exclusively associated with OCD symptoms or whether it could be related to different impaired processes.

### N200

The N200 shows a similar topography and estimated source localization (ACC) as ERN (Van Veen and Carter, 2006). This ERP component appears between 150–400 ms after stimulus presentation (Ciesielski et al., 2011) and appears to be involved in situations in which responsive conflict is high (Kopp et al., 1996b; Liotti et al., 2000; Wang et al., 2000; Nieuwenhuis et al., 2003). In 2002, Van Veen and Carter (2002b) argued that the post-stimulus latency window of the N200 indicates that it occurs prior to the response in correct conflict trials (Yeung et al., 2004). This idea is consistent with the hypothesis that the N200 reflects conflict detection, thus suggesting cognitive activity before an action takes place. Several empirical findings argue for a cognitive conflict control-related N200 component that originates in the prefrontal dorsal and ACC regions (Gratton et al., 1988; Kopp et al., 1996a; Heil et al., 2000; Yeung et al., 2004; Van Veen and Carter, 2006).

In a variety of studies employing different tasks, the frontal-central N200 has been found to be largest under high conflict conditions (Kopp et al., 1996b; Van Veen and Carter, 2002a). An example is the Go/No-Go task, which creates competition between generating and withholding a response. Many studies have identified an enhanced N200 component in no-go trials (Pfefferbaum et al., 1985; Kok, 1986; Eimer, 1993). The view that an increased N200 amplitude in no-go trials is an electrophysiological correlate of conflict monitoring by the ACC is consistent with other proposals that the association of the N200 component with other tasks (e.g., Eriksen flanker task) is greater during incongruent trials than congruent trials (Kopp et al., 1996b; Liotti et al., 2000).

Several studies of somatosensory (Shagass et al., 1984a,b, 1988), auditory (Towey et al., 1990) and visual modalities (Ciesielski et al., 1981; Beech et al., 1983) have discovered evidence of abnormal ERP features in OCD patients that are consistent with increased cortical arousal. To analyze the occurrence of deviant sensory and cognitive information processing in OCD patients, Towey et al. (1990, 1993) employed an oddball paradigm. They found that OCD patients showed greater amplitude negativity than normal controls with respect to N200 activity. Additionally, ERP component modulation has been associated with longer latencies: normal controls showed longer N200 latencies during the difficult task compared to the easy one, but patients did not. These abnormalities have been interpreted as overfocused attention with frontal lobe region hyperactivation.

**Figure 4:**
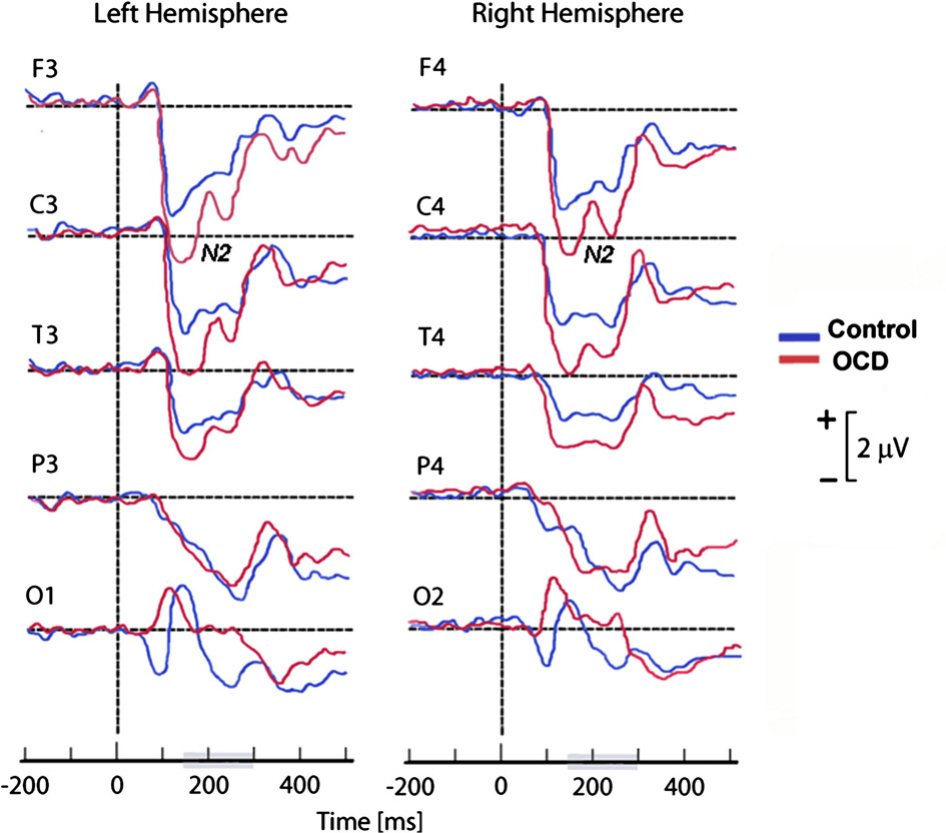
**Grand mean ERPs of the task epoch reflecting the cortical response to high-conflict stimuli**. The high-conflict N200 showed a higher amplitude in both groups of subjects. The differences between groups were significant in the prefrontal, central and temporal scalp locations, but not in the posterior brain regions. Reproduced with authorization from Clinical Neurophysiology (Ciesielski et al., 2011).

As frontal areas are presumably involved in the inhibition system (Rieger et al., 2003), another hypothetical explanation of the discordance between abnormally high cortical activation and faster or more accurate performance in OCD patients (Ciesielski et al., 2011) may be related to inhibitory control difficulties. In an attempt to investigate whether the N200 was associated with abnormal top-down cognitive control, Ciesielski et al. (2011) performed ERP amplitude measurements in four clusters of brain regions: the prefrontal, central, temporal, and fronto-polar regions. To correlate anterior brain N200 components with inhibitory attentional control, they applied a Stroop task requiring effective top–down monitoring and conflict evaluation. The most important result of this study was the particularly abnormal increase of activity observed in the prefrontal region of OCD patients (see Figure 4). Ciesielski et al. proposed that high activation of these brain areas could be a sign of an adaptive state of attentional readiness to sustain effective top–down inhibitory control in the context of interfering internal and external distractors. An enlarged N200 may be due to the patients' effort to maintain attention and normal performance.

In summary, the N200 is generated in the ACC and other areas of the prefrontal cortex (Towey et al., 1990, 1993; Nieuwenhuis et al., 2003; Ciesielski et al., 2011), and its role in action monitoring can be directly related to the FSMOCD. It has been proposed that the increased N200 amplitude is related to response conflict monitoring, and this effect is evident in incongruent trials (Kopp et al., 1996b; Liotti et al., 2000; Yeung et al., 2004). However, several findings suggest that higher N200 activity might reflect an increase in the inhibitory control of prefrontal areas (Ciesielski et al., 2011). Although there is a long way to go before the N200 can be used as a neurobehavioral tool for cognitive information processing, there is appealing evidence that the N200 amplitude is enhanced in OCD patients (Towey et al., 1993; Ciesielski et al., 2011). Nevertheless, further studies should be performed to investigate the exact role of the N200 in higher cognitive functions and the possible relationship between top–down attentional control and the performance of OCD patients on difficult conflict tasks.

### P600

**Figure 5:**
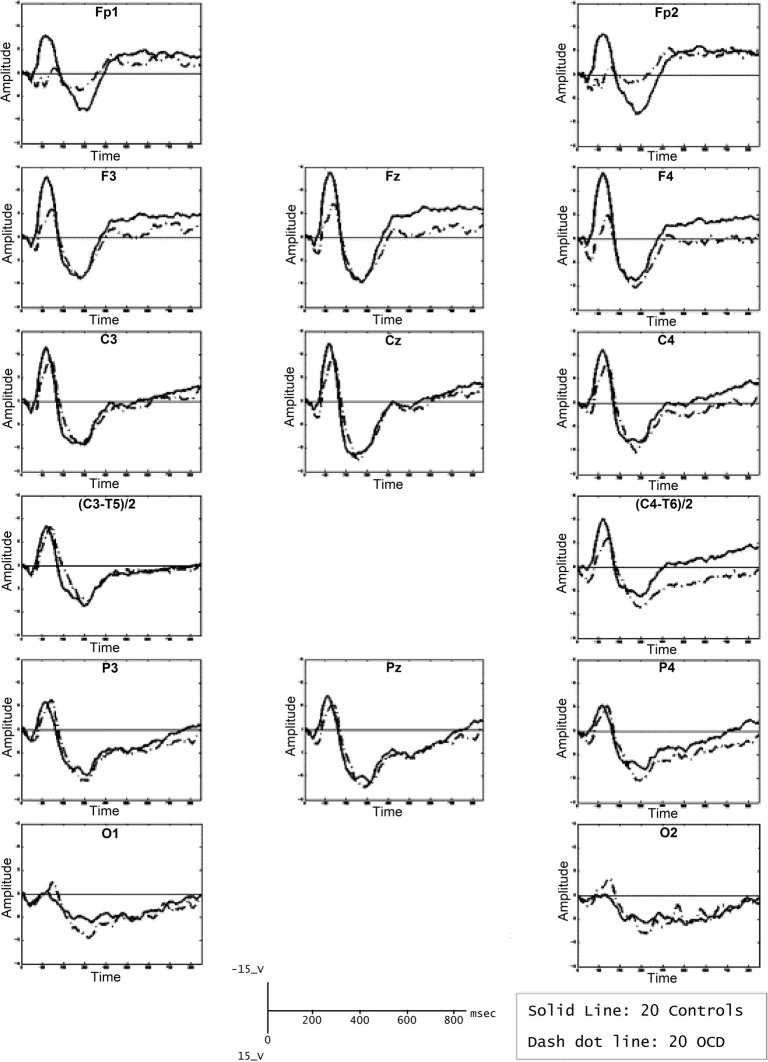
**Average of P600 waveforms of OCD patients (dotted line) and controls (solid line)**. In this experiment, subjects were presented with a computerized version of the digit span subtest of the Wechsler Adult Intelligence Scale. A sound was presented, after which subjects were asked to memorize the following numbers. Afterward, the signal tone was repeated, and the subjects were asked to recall the numbers. Reproduced with authorization from Psychiatry Research (Papageorgiou and Rabavilas, 2003).

The P600 is a positive wave that appears approximately 600 ms after stimulus presentation. The P600 might be related to WM because it has been considered as an index or second-pass parsing process. The P600 involves a wide fronto-basal network. While P600 estimation sources and fMRI co-recordings have shown involvement of prefrontal structures (Proverbio et al., 2009; Kompus et al., 2011; Maillard et al., 2011), fMRI recordings of the same paradigms eliciting a P600 and ERP studies in lesion subjects have shown involvement of both the frontal cortices and the BG (Friederici and Kotz, 2003). Compared with healthy controls, patients with OCD showed significantly higher P600 amplitudes in the right temporo-parietal area, which has been associated with obsessive traits (Papageorgiou and Rabavilas, 2003). Another interesting aspect is the increased latencies discovered in the centro-parietal area; this prolongation might suggest that OCD patients perform more slowly across all neuropsychological tests, which has been attributed to distracting obsessive thoughts during testing. These findings are consistent with the hypothesis that P600 latency increases as a function of the difficulty of response selection (Falkenstein et al., 1993, 1994). Although additional studies should be performed, the current results support the existence of an abnormal second-pass parsing in information processing in OCD patients (see Figure 5), which can be observed in WM tasks that elicit a P600 (Papageorgiou and Rabavilas, 2003; Kalatzis et al., 2005).

The longer latencies and higher amplitudes of the P600 may be related to the neuropsychological dysfunction found in OCD patients regarding working memory, which could be caused by patients' inability to suppress intrusive thoughts that prevent them from correct information processing and consequently result in low performance. These findings connect the P600 to the FSMOCD due to the fronto-basal network alteration implied in this model and its connection to information processing deficits related to working memory dysfunction.

## Discussion

This review aimed to integrate evidence from ERP, neuropsychological and neuroimaging studies on OCD and to analyze the relationship between these findings and the extended FSMOCD. To this end, we first described the most consistent neurocognitive deficits related to this disorder that have been reported in the neuropsychological literature. Next, we analyzed the neuroimaging findings associated with these neurocognitive dysfunctions. Finally, we focused our attention on individual ERP components (ERN, N200, and P600) that have been found to be impaired during electrophysiological investigations of OCD patients.

### The contribution of ERP research and its convergence with neuropsychology and neuroimaging studies

OCD presents a highly variable neurocognitive profile regarding neuropsychological and neuroimaging assessments. This finding is not unexpected given the heterogeneity of symptoms and high co-morbidity involved in OCD (Kuelz et al., 2004; Chamberlain et al., 2005; Menzies et al., 2008). OCD can be better understood at a system or network level (Menzies et al., 2008). Nevertheless, ERP research provides systematic and replicated results supported by different manipulations: OCD presents enhanced ERN and N200 responses during conflict tasks as well as an enhanced P600 during WM tasks. These combined results suggest enhanced ACC/OFC and reduced DLPF/parietal sites. Thus, the extended version of the FSMOCD including ACC/OFC/BG networks in connection with DLPC/parietal sites is supported by the ERP results reviewed here.

**Figure 6:**
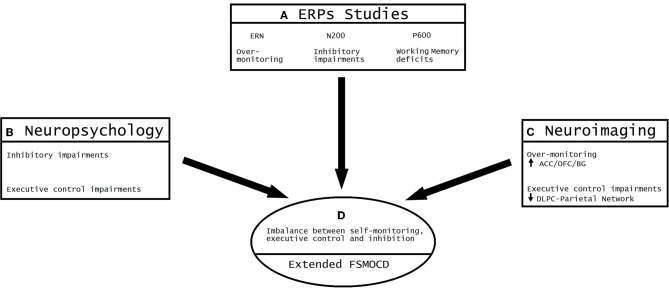
**Convergent evidence from ERP, neuroimaging and neuropsychology results: the imbalance between over-monitoring and inhibitory impairments**. **(A)** The most consistent findings of ERP studies on OCD suggest the existence of excessive over-monitoring (an enhanced ERN amplitude), inhibitory impairments (N200 enhancement) and working memory deficits (P600 enhancement). **(B)** The neuropsychological results show both inhibitory impairments (assessed through multiple tasks) and executive control deficits (planning, working memory and attentional set-shifting). **(C)** Neuroimaging convergence. Over-monitoring of the conflict system (increased activity of ACC/OFC/BG) and executive impairments (reduced activation of the DLPC-parietal network). **(D)** Convergent model of the extended FSMOCD proposing an imbalance between self-monitoring, executive control, and inhibition, indexed by an overactive ACC/OFC/BG circuit and impaired DLPFC/parietal-related network.

According to this convergence (see Figure 6), OCD can be understood as a model of unbalanced self-monitoring and inhibition. In this model, monitoring and inhibition appear to be crucially affected, besides executive impairments (especially WM and planning). Despite the heterogeneous results related to neuropsychological impairments, deficits in inhibition assessed during different tasks are the most consistent results (Chamberlain et al., 2005). Other deficits appear to be secondary to EF impairments, especially altered cognitive strategies (Kuelz et al., 2004) related to action monitoring. Among the neuroimaging findings, the most replicated result is overactivation of the OFC/ACC/BG, reflecting abnormal inhibition and monitoring. In addition, other areas outside the classic cortico-striatal system appear to be affected by executive functions and WM (DLPC/Parietal sites). Finally, ERP research evidenced an overactivated error detection system and inadequate processing of the motivational significance of errors and reward processing, both of which are related to OFC/ACC/BG interactions. Importantly, the results of ERP studies (given the modulation of ERN and N200) support the existence of an early influence of automatic monitoring system, possibly prior to later executive dysfunctions. The P600 findings suggest a later imbalance of the fronto-striatal system in connection with the DLPC/parietal sites (Menzies et al., 2008; Milad and Rauch, 2012). This convergence of different methods has not been noted in previous reviews because current ERP research is not integrated with neuroimaging and neuropsychology outcomes.

Neuroanatomical models of both monitoring (Kopp et al., 1996b; Liotti et al., 2000; Wang et al., 2000; Nieuwenhuis et al., 2003; Heekeren et al., 2008; Menzies et al., 2008) and inhibition (Chamberlain et al., 2005; Haber and Knutson, 2010; Milad and Rauch, 2012) involved the FSMOCD. OCD patients exhibit an impaired ability to control, monitor, and inhibit intrusive thoughts, urges, feelings, and behaviors (Milad and Rauch, 2012). In fact, it has recently been proposed that there is cross-talk between the FSMOCD, inhibition/control, and the symptoms of impulsivity and compulsivity (Robbins et al., 2012). The impaired cognitive and emotional regulation and control observed in OCD involves hyperactivation of the ACC, OFC, and BG (Taylor and Liberzon, 2007). In the present model, this monitoring and inhibition imbalance is explained by the combination of an excitatory role of the BG (associated with cognitive or motor actions without volitional control) and inhibitory over-activity of the OFC as well as excessive monitoring of the ACC to withhold BG excitatory impulses. This imbalance would interact with the reduced activation of the parietal-DLPC, leading to executive dysfunction. Although this model appears to fit better with a dimensional spectrum of OCD than a clear psychiatric nosology, this approach is consistent with recent consideration of OCD as a group of obsessive-compulsive spectrum disorders (Phillips et al., 2010; Robbins et al., 2012).

#### Inhibitory control in OCD impairment

Failures to inhibit automatic cognitive or motor processes are important characteristics of OCD (Abbruzzese et al., 1995a, 1997; Gross-Isseroff et al., 1996; Cavedini et al., 1998; Moritz et al., 2001b; Spitznagel and Suhr, 2002; Aycicegi et al., 2003). It has been proposed that the role of the OFC area might be inhibitory control of automatic processes (Tremblay and Schultz, 1999; Elliott et al., 2000; Tremblay and Schultz, 2000; Hikosaka and Watanabe, 2004), which are considered to be modulated by the BG (Zald and Kim, 1996).

Response inhibition has also been assessed in ERP studies (N200 amplitude enhancement in OCD patients). This higher N200 amplitude could be a sign of attention readiness to sustain effective top–down inhibitory control to avoid the interference of distracters. Thus, convergent evidence highlights a core inhibitory control impairment in this disorder (Chamberlain et al., 2005; Haber and Knutson, 2010; Milad and Rauch, 2012). This tendency of OCD patients to fail in controlling automatic behaviors would facilitate the generation of obsessive thoughts and compulsive actions.

#### Monitoring and control impairments in OCD

A dysfunctional action-monitoring system has also been reported to be a core process underlying some of the characteristic symptoms of OCD (Ursu et al., 2003). Several neuroimaging studies (Swedo et al., 1989; Machlin et al., 1991; Perani et al., 1995; Carter et al., 1998; Botvinick et al., 1999; Van Veen and Carter, 2002a) have reported that the ACC area is hyperactivated in OCD patients. The ERP component directly associated with monitoring this process is the ERN. Although ERP monitoring has a low spatial resolution, there are several lines of evidence from different types of studies that support the hypothesis that ERN is mainly generated in the ACC. Additionally, an increased ERN amplitude has been reported in OCD patients compared to control subjects.

These results lead to different functional explanations for ERN and the ACC depending on the different available theories. Despite the differences between these theories with regard to the monitoring roles of the ACC and the ERN, other electrophysiological studies involving the N200 component (Van Veen and Carter, 2002a; Nieuwenhuis et al., 2003; Yeung et al., 2004) and neuroimaging (Van Veen and Carter, 2002a; Ursu et al., 2003) findings provide convergent information that support the conflict theory. Although some studies have suggested that the N200 is associated with response inhibition (Ciesielski et al., 2011), others propose the complementary hypothesis that this component might be related to the conflict response monitoring system (Van Veen and Carter, 2002a; Nieuwenhuis et al., 2003; Yeung et al., 2004). This view provides a unifying account of the N200 components observed in a variety of experimental tasks, and it is consistent with the evidence that links ACC activity to evaluative aspects of cognitive control (Carter et al., 1998; Nieuwenhuis et al., 2003). This abnormal cognitive process is not reflected in neuropsychological attention tasks, where OCD patients exhibit the same results as healthy controls (Zielinski et al., 1991; Hollander et al., 1993; Martin et al., 1993; Aronowitz et al., 1994; Berthier et al., 1996; Cohen et al., 1996; Savage et al., 1996; Milliery et al., 2000; Okasha et al., 2000; Moritz et al., 2002).

Agam et al. (2011) reported a contradictory result regarding the role of the ACC in ERN, as they observed activation of the posterior cingulated cortex using both fMRI and ERN measures. Nevertheless, this finding appears to be explained by differences in the experimental manipulations applied. They did not use a classical error or gambling task (go/no go, flanker, and Stroop), but rather, used an antisaccade task. This task appears to be mediated by very different brain areas, such as the thalamus (Peterburs et al., 2011). Importantly, no study addressing ERN in OCD using an antisaccade task has been reported. Moreover, following studies applying simultaneous ERP/fMRI recordings with classical error paradigms do not replicate the Agam results (Donamayor et al., 2011; Edwards et al., 2012). Conversely, they observed strong ACC activation in response to errors in both fMRI and ERN. Involvement of the ACC in monitoring is a very consistent result in both neuroimaging (Bush and Shin, 2006) and ERP reports (Taylor et al., 2007) using conflict tasks. ACC lesions produce robust effects on ERN (Stemmer et al., 2004; Hogan et al., 2006). The same result is obtained using virtual lesions (TMS) (Rollnik et al., 2004). Intracerebral studies providing direct intracranial sources confirmed the involvement of the ACC in ERN (Brazdil et al., 2005; Jung et al., 2010). In conclusion, neuroimaging, electrophysiological and neuropsychological investigations provide support for the ERN generation on the ACC.

#### Executive functions and their relationship with monitoring and inhibition

Evidence from neuroimaging studies in OCD patients showed decreased responsiveness of the DLPFC in EF tasks and a decreased volume of this region. The DLPFC has been associated with planning abilities (Menzies et al., 2008), WM processes and set shifting, among other executive functions. The parietal cortex (Cabeza and Nyberg, 2000; Culham and Kanwisher, 2001) is also important in higher order cognitive tasks, such as WM and attention shifting. A study (Papageorgiou and Rabavilas, 2003) comparing OCD patients and healthy controls found that the amplitude of the P600 component in the right temporo-parietal area was significantly higher in OCD patients. This area is believed to contribute to the second-pass parsing process related to WM. According to this hypothesis, the longer latency and higher amplitude of the P600 may be related to the neuropsychological dysfunction in WM. Several studies (Cavada and Goldman-Rakic, 1989; Romanski et al., 1997; Roberts et al., 2007) have demonstrated a connection between parietal regions and the DLPFC, and both regions contribute to the dorsolateral prefrontal-striatal circuit. Neuroimaging results showing hypoactivation of this circuit and findings related to the P600 component provide consistent evidence of impairment in OCD patients with respect to encoding, organizing, planning, and implementing effective strategies. In addition to the overactive conflict response monitoring system, these deficits could be related to doubt and slowness in this disorder.

#### Other neuroanatomical models of OCD

Other studies have examined the potential influence of additional brain structures in the neuropathology of OCD that were not assessed here. Several researchers support fronto-striato-limbic models of OCD (Simon et al., 2010; Milad and Rauch, 2012) that attribute a specific role in mediating the anxiety symptoms to the amygdala and associated para-limbic regions. The amygdala is extensively connected, both anatomically and functionally, to the OFC and ACC (Carmichael and Price, 1995; Rolls, 1999; Cavada et al., 2000) and ascribes an affective function to the fronto-striatal network (Lawrence et al., 1998; Phillips et al., 2003). The amygdala also projects strongly to the mediodorsal nucleus of the thalamus (Amaral et al., 1992), the final relay station before the OFC/ACC/BG loops project back to the cortex (Alexander et al., 1986; Middleton and Strick, 2001), and it is therefore critically positioned to influence the output of these loops (Maia et al., 2008). The amygdala plays a crucial role in mediating normal fear and anxiety (Ledoux, 2000, 2007; Phelps and Ledoux, 2005) and contributes to anxiety disorders (Rauch et al., 2003; Bremner, 2004; Miller et al., 2005). Nevertheless, the role of this region in OCD remains to be elucidated. One recent study found increased amygdala activation in patients with OCD during active responses to emotional faces (Cardoner et al., 2011). Another study reported that although amygdala hyperactivation was observed in response to symptom-provoking stimuli, such hyperactivation was unrelated to the OCD symptoms (Simon et al., 2010). Moreover, studies of amygdala volume in adults with OCD have yielded inconsistent findings [bilateral reduction: amygdala (Szeszko et al., 1999) and left increase (Kwon et al., 2003b)]. Thus, it is important to note that the role of the amygdala in the pathophysiology of OCD requires further investigation.

Another question that remains is whether the temporal lobe may also be involved in the pathogenesis of OCD (Maia et al., 2008; Morein-Zamir et al., 2010). Several studies have reported anatomical abnormalities in the superior temporal gyrus (Choi et al., 2006; Shin et al., 2007; Yoo et al., 2008). Recent meta-analyses have demonstrated anatomical and functional changes in the medial temporal lobes of OCD patients (Menzies et al., 2008). One possibility is that the temporal lobe (in particular, the superior temporal gyrus) is involved in OCD via its connections with the regions of the striatum that are part of the OFC/ACC/BG loops (Alexander et al., 1986). Additional research is needed to test this hypothesis.

Finally, abnormalities in other areas closely related to the FSMOCD, including the hippocampus and the insula have also been reported. Voxel-based morphometry studies have highlighted abnormalities in the insula (Pujol et al., 2004; Valente et al., 2005; Yoo et al., 2008), and volume reductions have been reported in the hippocampus (Kwon et al., 2003b) in adults with OCD, supporting the existence of widespread abnormalities across the brain (Menzies et al., 2008). The insula is interconnected with both the OFC and the ACC (Mesulam and Mufson, 1982; Ongur and Price, 2000; Ibanez et al., 2010a; Ibanez and Manes, 2012; Couto et al., 2012), suggesting that more distributed large-scale brain systems may be involved in OCD (Menzies et al., 2008). Further research is required to establish whether the OCD results regarding the insula and hippocampus can be directly related to the FSMOCD.

## Limitations and further studies

ERP studies have often been applied to paradigms that indirectly reflect specific cognitive functions in OCD. Several neuroimaging studies (Breiter et al., 1996; Adler et al., 2000; Mataix-Cols et al., 2002; Shapira et al., 2003; Nakao et al., 2005) have been carried out using symptom provocation paradigms that more directly reflect the physiological reaction to stimuli that provoke certain symptoms. Although OCD is usually a chronic disorder, anxiety is experienced only when the individual encounters stimuli that trigger obsessive fears. Future ERP studies should employ symptom provocation paradigms, which could shed light on the neurological mechanisms that take place during the specific moment at which symptoms occur.

Another general limitation is co-morbidity. The discrepancies among different hypotheses could be partially attributable to the heterogeneity of this disorder and, more specifically, to differences between patient subgroups (Tallis, 1997b). Similar caveats for OCD neuroimaging studies related to methodological issues (sample size, multiple corrections, non-selective data analysis) are a topic for further consideration. Finally, it is worth noting that most OCD patients have an extensive history of medication use. Even if the global effects of these treatments on the reported findings remain unclear, there is interesting evidence from neuropharmacological investigations suggesting that various substances alter ERP components.

## Conclusions

The role of ERP as a methodology that connects OCD symptomatology with the fronto-striatal model has numerous implications. Perhaps the most important of these implications is the linkage of ERPs (ERN and N200) with action monitoring and inhibition, particularly because they are modulated by the ACC, OFC, and BG, which are intimately linked to OCD.

ERP studies offer several advantages. One of the most relevant is a temporal resolution that allows a more precise analysis of different stages of cognitive processes during the performance of a motor or cognitive task. Another advantage is its lower cost compared to other techniques, such as neuroimaging. The integration of neuropsychological studies, neuroimaging techniques and ERP findings might become a powerful strategy for obtaining more precise and complete knowledge of disorders such as OCD. This integrative approach could also be useful in analyzing the possible role of conflict monitoring and inhibition (ERN, N200) as well as executive functions such as WM (P600) as biomarkers or endophenotypes of this disorder.

### Conflict of interest statement

The authors declare that the research was conducted in the absence of any commercial or financial relationships that could be construed as a potential conflict of interest.
